# Damage Identification in Structural Health Monitoring: A Brief Review from its Implementation to the Use of Data-Driven Applications

**DOI:** 10.3390/s20030733

**Published:** 2020-01-29

**Authors:** Diego A. Tibaduiza Burgos, Ricardo C. Gomez Vargas, Cesar Pedraza, David Agis, Francesc Pozo

**Affiliations:** 1 Departamento de Ingeniería Eléctrica y Electrónica, Universidad Nacional de Colombia, Cra 45 No. 26-85, Bogotá 111321, Colombia; rcgomezv@unal.edu.co; 2 Escuela de Tecnologías de la información y la comunicación, Politécnico Grancolombiano Institución Universitaria, Bogotá 111321, Colombia; 3 Departamento de Ingeniería de Sistemas e Industrial, Universidad Nacional de Colombia, Cra 45 No. 26-85, Bogotá 111321, Colombia; capedrazab@unal.edu.co; 4 Control, Modeling, Identification and Applications (CoDAlab), Departament de Matemàtiques, Escola d’Enginyeria de Barcelona Est (EEBE), Universitat Politècnica de Catalunya (UPC), Campus Diagonal-Besòs (CDB), Eduard Maristany, 16, 08019 Barcelona, Spain; david.agis@upc.edu (D.A.); francesc.pozo@upc.edu (F.P.)

**Keywords:** data-driven algorithms, damage identification, structural health monitoring, sensors

## Abstract

The damage identification process provides relevant information about the current state of a structure under inspection, and it can be approached from two different points of view. The first approach uses data-driven algorithms, which are usually associated with the collection of data using sensors. Data are subsequently processed and analyzed. The second approach uses models to analyze information about the structure. In the latter case, the overall performance of the approach is associated with the accuracy of the model and the information that is used to define it. Although both approaches are widely used, data-driven algorithms are preferred in most cases because they afford the ability to analyze data acquired from sensors and to provide a real-time solution for decision making; however, these approaches involve high-performance processors due to the high computational cost. As a contribution to the researchers working with data-driven algorithms and applications, this work presents a brief review of data-driven algorithms for damage identification in structural health-monitoring applications. This review covers damage detection, localization, classification, extension, and prognosis, as well as the development of smart structures. The literature is systematically reviewed according to the natural steps of a structural health-monitoring system. This review also includes information on the types of sensors used as well as on the development of data-driven algorithms for damage identification.

## 1. Introduction

Ensuring the proper performance of all elements in a structure is a priority for designers and users. In most cases, continuous monitoring can detect damages at an early stage can prevent potential accidents and catastrophes that result from inadequate inspection or damages to the evaluation process. Structural health monitoring (SHM) involves the use of continuous monitoring using sensors that are permanently attached to the structure, together with algorithms related to the damage-identification process. There are several advantages associated with the use of an SHM system, some of which are listed below:

- the continuous monitoring of the structure since sensors are a part of it;
- the possibility of real-time damage detection;
- the possibility of using sensor or actuator networks;
- robust data analysis that can provide relevant information about the damage;
- an automated inspection process to reduce the number of unnecessary maintenance tasks, thereby improving the economic benefits; and
- operational and environmental evaluation conditions.

Although SHM is still a developing area—as evidenced by the rapid increase in the number of research works and publications—research has been ongoing for the past 23 years [1]. Both the benefits in the above list and the advances in computation and data science applications motivate the continually rising interest in structural health-monitoring applications.

**Figure 1:**
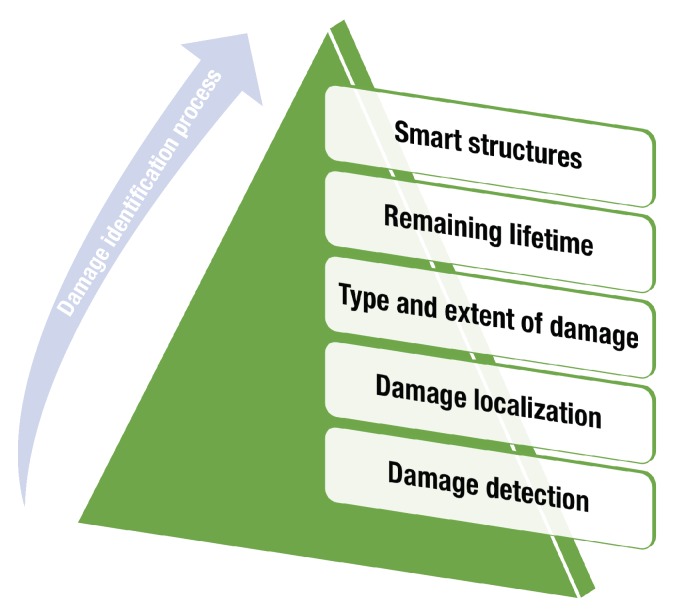
Damage identification levels.

Different levels of damage diagnosis in SHM were proposed by Rytter [2]. These levels are defined on the basis of the information that can be obtained during the damage identification process. In general, *damage detection* is the first level of damage diagnosis and can provide information about irregular behavior of the structure that, in some cases, can be regarded as possible damage [3]. After damage detection, damage localization (Where is the damage?), damage classification (What kind of damage does the structure have? damage extention) and damage prognosis (What is the remaining useful life of the system?) are considered, as shown in Figure 1.

Different algorithms and methodologies have been developed for each level of the damage identification process, including the management of historical information on the functioning of the structure, and they often use different sensors and actuators, materials, and configurations. Some of the works available in the literature have focused on problems related to a single level of SHM [4], a specific application [5], a specific technique [6], or a certain type of sensor for inspection [7]. For example, at the level of damage detection, aspects such as sensor locations and the use of wireless sensor networks [8] as well as the use of specific kinds of sensors or sensor networks, such as microelectromechanical systems (MEMS) [9], accelerometers, optical fibers [10], vibration sensors [11], and pressure-based sensors [12] have been addressed. Similarly, this level has been tackled using different techniques, as shown throughout this review. Neural networks [13,14,15], modal analysis [16], bio-inspired algorithms [17], non-probabilistic methodologies [18], and time series analysis [19,20,21] are among the main techniques that are used. The autonomy of SHM systems has also been addressed through the possible ways in which they obtain energy [22]. Other works have examined the use of mechanical energy from different sources, such as thermal energy, wind energy, solar energy, electromagnetic sources, or hlRF antennas [23].

Other state-of-the-art reviews have concentrated on SHM applications in different areas, such as the aeronautical industry [22], wind generation [24], civil engineering applications [25], and naval engineering [26]. It is also possible to find review papers that are oriented toward the development of SHM methodologies with guided waves [27,28] and the use or integration of the Internet of Things (IoT) [29] in SHM applications.

This review is focused on the use of data-driven methodologies for all levels of the damage-identification process. This work is organized as follows: Section 2 is devoted to the description of the SHM process, including different approaches to analyzing SHM systems and the variables that are identified in the operational and environmental conditions that affect damage identification. In Section 3, the SHM process and its implementation are described. The implementation of SHM is included in Section 4, along with information about some of the elements of SHM systems such as data acquisition, sensors and actuators, and preprocessing strategies. This section also presents works on the decision-making process. Finally, conclusions drawn from the reviewed literature are summarized in Section 5.

## 2. Description of the SHM Processes

Several definitions have been used to define damage; however, one of the most accepted definitions was given by Farrar and Worden [30]:

“Damage is defined as changes to the material and/or geometric properties of these systems, including changes to the boundary conditions and system connectivity, which adversely affect the system’s performance.”

This definition indirectly implies that all SHM applications, including online monitoring, require an adequate sensor network system to evaluate possible changes in the structure that can affect its proper performance. Usually, the sensibility of the SHM system is associated with good interaction between the structure and the sensors. For this reason, it is very important to select appropriate sensors to be installed by considering the material of the structure to inspect, the variables to sense or measure, and the information to obtain for damage identification.

For SHM applications, increasing the reliability of the forecasts or predictions and the damage identification process is a fundamental task in the implementation of these approaches in the industry. Therefore, the number of false positives detected because of noise or the acquisition process must be minimized. For this purpose, some reliability indices have been proposed. For instance, reliability analysis has been based on the estimated distributions of dead, live, and wind loads in long-span bridges [31].

Failures caused by inconsistencies between the capturing techniques, the information of the sensors, the processing of the information captured, and the analysis of data for the forecast can affect the results obtained from the algorithms or the methodologies used in the damage-identification process [17,32].

In SHM, several current approaches to evaluating the integrity of a structure at any moment under different operating conditions are based on measuring changes in the mechanical, physical, or chemical behavior of the structures under inspection. As illustrated in the following sections, various techniques have been implemented to capture and analyze information from a sensor network that is installed and used for continuous monitoring. As discussed later in this paper, the analysis carried out in some of the current methodologies not only aims to identify possible existing damages but also is used in the development of forecasts about the future behavior of the inspected materials and structures.

In general, SHM developments can be classified into two large groups [30]: *model-based* approaches and *data-driven* approaches. In the first type of approach, theoretical information or data acquired from the structure are used to build a mathematical or physical model to predict the behavior of a structure in different scenarios and with different variations in operational and environmental conditions. Model-based approaches make frequent use of finite element analysis (FEA) [33]. The second approach to damage identification relies on the analysis of data acquired directly from the structure. In general, this analysis can be performed under the pattern recognition paradigm [30]: that is, data obtained from the healthy structure are used to build a pattern and data from the structure during the inspection process are used to determine its current state by comparing some features obtained from the inspection data with the baseline. Both types of methods—model-based and data-based—can be applied at every level of the damage-identification process in Figure 1. This means that, currently, there are different strategies that consider a single level or multiple levels of the damage-identification process. Implementation at particular levels delivers specific results that may be the end of the process, depending on the application. It is also possible, for example, to build a solution of which the intended scope is limited to the detection and location of damages. In other cases, the expected scope is related to the implementation of the first four levels, thus determining the remaining useful life of the structure under inspection. However, it is clear that all levels present an incremental approximation since the implementation of a particular level requires the performance of the previous levels in the pyramid in Figure 1.

In terms of applications, different engineering associations and events perform analyses of SHM benchmarks, which present a set of solutions applicable to specific scenarios, such as the American Society of Civil Engineerings with the IASC-ASCE SHM Benchmark Study [34], and the international association for bridge maintenance and safety (IABMAS) conference decisions in Kyoto at 2014 with the benchmarks from the University of Central Florida and the Drexel University, and other studies that guide the instrumentation of civil structures. Similarly, other works, such as that in Reference [35], have developed a classification scheme for benchmarking methods in use that include simulation and implementation.

Since structures are subjected to operational and environmental variations during their use, it is necessary to consider these variables in the damage-identification process. In fact, these variations can be regarded as a disturbance in some SHM algorithms and need to be considered in order to reduce the possibility of a poor identification process. The subsequent sections provide a brief review of some works that have addressed operational and environmental conditions. All these works have been arranged using the four-step approach presented by Farrar [36].

## 3. SHM Implementation

Farrar [36] suggested that SHM developments must comply with economic, environmental, operational, and temporary restrictions, among others. These factors must be analyzed before proposing an SHM system as a solution. From this point of view, some works and considerations focused on these topics are summarized here.

### 3.1. Economic Justification

Before undertaking the development and application of a structural inspection scheme, it is important to ensure that the solution reached is coherent with respect to (i) the resources it will require; (ii) the response time; (iii) the margin of error that is allowed; and, in general, (iv) compliance with the operational conditions and constraints in the application of this kind of scheme.

Most applications of damage-detection schemes can reduce maintenance costs and the frequency of inspections. These detection schemes result in an increase in the remaining lifetime of the structures. For example, in the aeronautical industry, the utility of SHM is reflected by the reduced periodical revision times, the increased availability and safety of aircraft, and the decreased costs of scheduled repairs [37].

Industries such as power generation have reported substantial costs associated with the repair of turbines. These expenses are significantly increased in offshore platforms [38]. Therefore, the use of SHM as a tool to prevent sudden damage yields essential benefits in this industry [39].

One of the challenges associated with the use of SHM applications in industries is the initial cost of implementing the technology and of ensuring its overall reliability. In this context, the developments such as new transducers and low-cost sensor networks for inspecting large structures, together with the benefits gained by these low-cost sensors compared with the cost of visual supervision and traditional inspection methods in applications such as the inspection of civil structures [40], have resulted in the rapid expansion of the use of SHM. As stated previously, this expansion has been mainly driven by the economic advantages of its use and its fast implementation [41], with the clear and direct consequence of money saved in the long term.

### 3.2. Operational and Environmental Conditions

Structures in operation are subjected to the influence of operational and environmental conditions. These conditions affect the structures causing degradation, aging, and damage [42]; they are also the cause of possible false detections in SHM systems, due to the sensitivity of the methods to operational and environmental variables (EOVs) [43,44]. For that reason, the influence of these variables must be considered in the development of a reliable SHM system [45].

Reducing the influence of operating and environmental conditions presents excellent opportunities in industries such as aeronautics, in which significant advances that reduce failures of the SHM system have been generated by solutions such as the reduction in environmental noise during data acquisition through the use of transducers with low-frequency digital–analog converters [46], combined with transmission systems based on fibers that reduce the noise in the transmission [47]. Damage detection and classification approaches that consider the effect of temperature with multivariate analysis are available in the literature. These approaches combine, for instance, the use of principal component analysis (PCA) and machine learning (ML) [48] or PCA and self-organizing maps (SOM) [49]. Other approaches consider different elements, such as the influence of the viscoelastic material properties of the adhesives used by sensors on SHM applications [50], the influence of temperature and surface wetting on the ultrasonic waves used for damage detection [51], and the relationship between feature extraction and data fusion. Proposed solutions include the use of sensor data fusion, PCA, and self-organizing maps to compensate for the undesirable effect of temperature on damage detection and classification [49], the use of optimal baseline subtraction [52], and the effect of elevated temperatures on the adhesive layers of piezoelectric transducers (PZTs). Other works have examined the effects of temperature on baseline impedance profiles and the use of a small subset of baseline profiles for certain critical temperatures to estimate baseline profiles for a given ambient temperature through interpolation [53]. Finally, the use of local density in self-organizing maps has also been considered for two-level clustering as a methodology to compensate for the effects of temperature [54] or the effects of extreme aeronautical environments on the use of wireless sensors for SHM [55].

Structures such as those used for marine platforms, which are exposed to variable environmental conditions, have shown failures in their systems of damage detection and location. In this context, Prendergast et al. [56] presented an analysis of the variation in eigenfrequencies of turbines under progressive scour. The work reported by Zhou et al. [57] demonstrated an SHM approach for marine platforms that accounted for the variability of the ocean environment, vibration, corrosion, marine currents, and the effects of collisions modeled using a transfer matrix. Their approach enabled the calculation of changes in the form of the structure and the differentiation of possible failures. Other closely related works have presented dynamic models of the behavior of this type of structure; these models include the relationship between ocean currents under the ocean and wind currents on the surface, which may affect the analysis of the captured data [39]. Oliveira et al. [58] show the implementation of modal analysis in turbines based on vibration, building strategies to reduce the influence of EOVs through statistical analysis.

In applications of SHM for civil structures, such as bridges or other buildings, the moving loads that affect these structures play a fundamental role in the determination of their lifetime. The use of vibration characteristics of vehicular bridges, which are analyzed as a source of vibration, has also been explored. For instance, methods such as mode shape as damage-detection indicator have been applied to analyze the behavior of a bridge [59]. Other proposals in this kind of structure seek to increase the reliability of operational modal analysis (OMA) concerning external phenomena, such as vibration [60], or the performance of estuaries in seismic areas [61].

Rainieri et al. [42] show the application of the second-order blind identification method (SOBI) to predict variations in natural frequencies, which makes it possible to compensate for the environmental influence on the use of OMA, this being a limitation of the PCA analysis. The implementation carried out requires a relatively low computational effort and obtaining a linear model between natural frequencies and unknown EOV sources.

### 3.3. Damage Definition

When using damag- detection techniques that are noninvasive and use sensors that are permanently installed on the structure, which is the case in SHM, the influence of the operation of the structure must be taken into account. This means that a small change in the structure can be detected as a different signal by the sensors, and the final result of the analysis can lead to a greater frequency of misclassification. In this case, the characterization of the behavior of the structure under different conditions and the definition of the influence of the operating conditions are critical tasks in the implementation of a reliable SHM system. It is very important to differentiate between a *normal* or an *acceptable* state and *damage* to determine whether the current state should be reported. Different studies have been carried out in this framework, with subjects ranging from the comparison of material [62] to the realization of failure models of structures.

The work in Reference [63] presents a list of modeling techniques for determining the existence of damage; they are based on the use of the smoothed finite element method (SFEM), focus on detection techniques based on high-frequency inputs, and use a number of tools for the definition of a fault.

### 3.4. Limitations

SHM has limitations associated with the capture and processing of data, the use of statistical models, and the interpretation of the results. As a consequence, ad hoc schemes must be generated for different SHM applications. Determining the limits of the application as well as the tools and the techniques involved can increase the certainty of the prediction.

Studies associated with nonlinear analysis methods have been reported in different works [64,65]. Similarly, methodological limitations caused by the propagation of linear excitation signals were described in Reference [66].

The inspection of structures using stationary cameras is an SHM practice that is widely used for civil structures. These procedures present challenges with respect to locating an optimal site at which to place the camera [67]. This problem, sometimes, is dealt with by using moving video systems. The work in Reference [68] introduced a solution to the limitations in the instrumentation of large civil structures. The proposed solution combined the use of georeferenced visual inspection systems with the use of technology such as the linear variable differential transducer (LVDT) and laser Doppler vibrometer (LDV).

Among the most important damage detection issues with any technique is its reliability, which is indispensable. Multiple works have reviewed this topic with respect to SHM [69,70,71]. Errors at the detection level have hindered the inclusion of SHM in industries such as the automotive and aeronautics sectors [72] and other mission-critical applications [73].

## 4. SHM Implementation Steps

**Figure 2:**
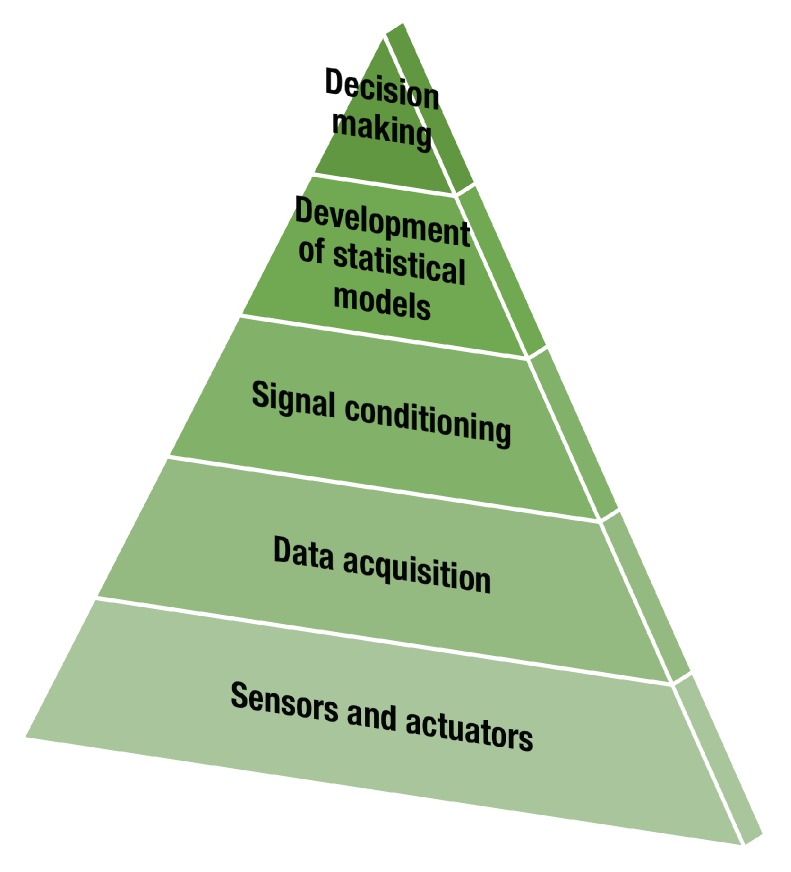
Development steps as a part of an structural health-monitoring (SHM) solution.

From a general point of view, the implementation of an SHM system requires four steps: (i) the definition of the sensor/actuator system to attach to the structure; (ii) the data acquisition system; (iii) the preprocessing step; and (iv) the development of statistical models. These four steps are represented in a pyramid in Figure 2. These steps allow for the generation of solutions to challenges in the different levels of the damage-identification process (Figure 1). An additional level can even be included to consider *smart* solutions in which the previous levels are evaluated to determine the best combination of multiple configurations to produce an optimal solution to the damage-identification task.

The lowest level in the pyramid in Figure 2, labeled “Sensor and actuator”, defines the first part of the hardware that will be used. The sensor/actuator system must be selected considering, among other factors, the type of variables to measure, the constraints related to physical or environmental conditions, and the type of information to obtain. As stated previously, this level corresponds to the definition of the type of sensor to be used, the configuration of the sensor network, and the possible actuators, if required [74]. These configurations must agree with aspects such as maximum operating frequencies, the extent or size of the damage to be identified, possible locations, characterization, and, in general, the physical limitations of the application.

The next level in the pyramid is related to data acquisition, also known as DAQ. Data acquisition refers to the way in which the signals generated by each sensor are obtained. At this level, some of the SHM system’s characteristics, such as cost, mobility, and scalability, need to be considered. The information acquired by the SHM system can be affected by aspects such as sensor configuration, operational and environmental noise, and any other event that differs from the initial setup of the system. Some of these problems must be resolved before performing any analysis on the generated information to generalize the techniques used for classification, identification, or recognition. This step corresponds to signal conditioning or preprocessing, and it can be performed by means of hardware devices, software algorithms, or both. In some cases, data can be corrupted or affected by a lossy transmission. These kinds of problems frequently appear, for instance, in applications that use wireless communication. This is the case for large structures [75]. Consequently, several works have addressed these issues to improve the reliability of data communication.

In most cases, the raw data obtained in the acquisition process require a data reduction step. For this purpose, a discrete wavelet transform (DWT) can reexpress the raw data by means of coefficients that decompose the data into so-called *details* and *coefficients* [76,77]. With this technique, it is possible to obtain a reduced representation of the original data. Similarly, techniques such as PCA [78] and independent component analysis (ICA) have demonstrated their versatility in this respect by reducing data to a few components by the criteria of the retained variance [79].

The step called “Development of statistical models” includes the use of data analysis tools to determine the existence of abnormalities in the instrumented structure and to characterize the possible sources of these anomalies. This step directly influences the costs of the solution in both economic and computational terms, and it undoubtedly affects the detection, location, and characterization of damages.

As the final step, it is possible to use decision tools that support the intervention processes and to define possible action routes to take. This step, “Decision making”, aims to reduce subjectivity in the development of the SHM process and to decrease the number of failures in the methods defined in the previous step. This step is not always considered or contemplated in an SHM solution.

### 4.1. Sensors and Actuators

From the perspective of SHM, damage detection requires the implementation of a set of sensors of which the main function is to capture information that can be used to determine the state of the structure under analysis [25]. Some inspection schemes use the propagation of a signal that may be produced by an actuator. The inspection schemes also depend on the types of transducers used and the kinds of signals propagated through the structure. As parts of a comprehensive sensor network, these sensors can obtain information from different parts of the specimen or structure under inspection. Similarly, sensor arrays require the use of various sources of excitation. In some cases, the actuators are located as close as possible to the sensors so that a transducer can serve as both an actuator and a sensor. This type of duality is exemplified by piezoelectric arrays [80].

The sensor/actuator system can be classified as *active* or *passive*. This classification depends on the source and whether the signals are propagated through the instrumented structure [81,82].

Passive methods only use sensors to detect variations in the received signals without the use of an external signal. The data obtained from these sensors can be used to detect structural abnormalities produced by the corrosion, deformation, or perforation of the materials [74,83]. Active inspection methods apply a known excitation and evaluate the data of the propagated signal. The excitation depends on the type of sensor and the interaction that is required within the structure.

#### 4.1.1. Excitation Methods

Active inspection methods can be classified as *linear* or *nonlinear* [74], depending on the propagation of the signals. Linear methods include the pitch-catch mode, in which an ultrasonic signal is applied by an actuator and the propagated signal is received by another sensor [84,85]; the pulse-echo technique, in which signal reflections are detected and the same actuator captures the signal that it transmitted [86,87]; and electromechanical impedance spectroscopy (EMIS), which is used mostly with piezoelectric sensors that monitor changes in the structural-mechanical impedance [88]. Other linear methods have also been developed [89,90].

Some linear methods use the propagation of Lamb waves in metal structures [91] with piezoelectric sensor arrays because of their directionality and low dispersion [92]. The propagation of this type of signal is used recurrently in the development of intelligent materials. In Reference [93], the dual optimization on PZT sensors was investigated to decrease the barrier imposed by the requirement of lines of the base (BF-SHM). The method used in the aeronautical industry varies the Lamb wave signals propagated, and an increase in the accuracy and reliability of the systems that use this type of signal was reported. Other works have focused on improvements in the process of identifying damages in structures. For instance, Li et al. [94] used the propagation of Lamb waves in isotropic materials to analyze the probability damage imaging (PDI) to improve the location and identification of damages in these materials.

Studies have also explored the use of nonlinear methods in the analysis of detection techniques that are based on the frequency of the propagated signal. For example, the work described in Reference [95] compared the results of stress experiments performed on different materials, and significant changes in the root-mean-square deviation (RMSD) analysis were found. Other works have aimed to develop multifrequency excitation systems, such as the system presented in Reference [74], in which the heterodyne principle was used to generate the signal to propagate in the structure. This work reported an increase in the probability of detecting small cracks.

Other works have investigated the use of adequate sources of excitation to improve the response of the implementation of SHM systems under environmental conditions. For instance, in Reference [96], arbitrary waves were propagated to reduce the influence of wind on the posterior analysis. As a result, a robust method was proposed for scenarios with noise, pollution, or exposure to adverse environmental conditions. With this type of analysis, works have reported the ability to decrease (relative to traditional methods) the computational load without affecting the detection [97].

#### 4.1.2. Types of Sensors

With the increased use of SHM approaches, new sensors have been developed that can improve the efficiency of detection, location, and characterization systems [25]. These developments aim to simultaneously reduce the power consumption and weight of the system, to resolve installation problems, and to improve operation facilities and the subsequent data analysis. The following subsections describe some of the different sensors used in SHM applications that are oriented toward the inspection of both metallic and composite materials.

**Figure 3:**
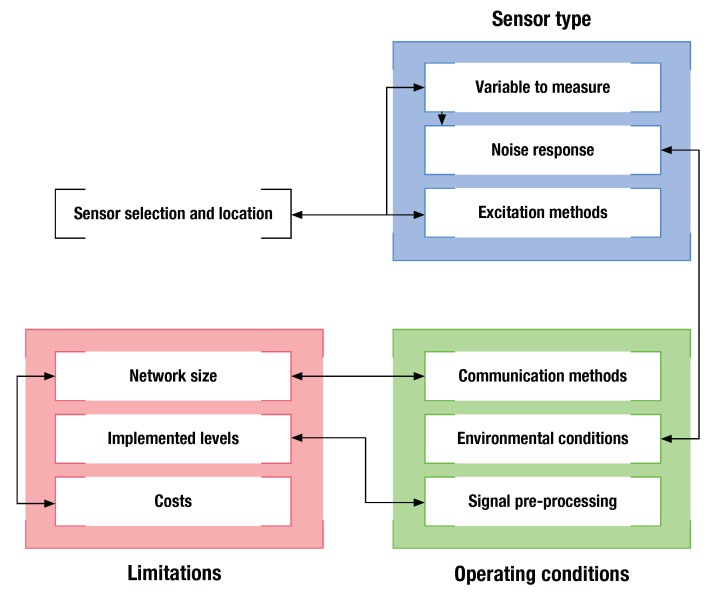
Sensor location and choice.

The choice and validation of sensors form one of the most important elements in this step. A correctly chosen sensor not only detects damages but also enables damage location, quantification, and classification [98]. Figure 3 describes some criteria to consider when selecting a sensor.

Sensors can be classified according to the physical variable that they sense [99] or the transduction principle on which they are based [98]. Table 1 includes some of these sensors and the variable that is usually inspected. The classification in Table 1 is used hereinafter, with an emphasis on their applications as well as the advantages and disadvantages of each type of sensor.

#### 4.1.3. Piezoelectric Sensors

Piezoelectric materials are built from ceramic and polymers, and they present the direct and inverse piezoelectric effect [100]. This is the reason that these materials are often used to make vibration-based sensors and actuators. The most frequently used materials in piezoelectric sensors are lead zirconate titanate (PZT) and barium titanate (BaTiO_3_). However, ferroelectric polymers, such as polyvinylidene difluoride (PVDF) and poly(vinylidene-co-trifluoroethylene) (P(VDF-TrFE)), are also used for their high piezoelectric and pyroelectric response levels [101].

An additional advantage of piezoelectric sensors is that they can be manufactured in different shapes, such as rectangular [102], longitudinal [103], and circular [104,105]. These piezoelectric sensors are also flexible and can be adapted to the shape of the structure at their installation locations. With the use of these sensors, it is possible to measure the vibration and to obtain information about different variables, such as deformation [106] or corrosion [107,108]. The literature also includes several applications with a wide range of frequencies, shape adaptation to structures [109], reduced size [110,111], and reduced phase changes [112], among other characteristics.

In terms of methods for inspection, piezoelectric sensors are frequently used in the development of electromechanical impedance (EMI) techniques. These techniques entail the evaluation of changes in the impedance of the sensor. EMI techniques are used in the inspection of civil structures, such as bridges, dams, and transport vehicles in aviation, as well as of trains and ships [113].

Corrosion is an interesting variable to measure and can be inspected using acoustic emission (AE). These sensors are placed in the structure and allow for the evaluation of different types of corrosion [114] as well as some loss of rigidity in the structures [115].

#### 4.1.4. Fiber Optics

Fiber optics are used in applications that require high precision and electromagnetic-interference immunity [116]. The principle underlying fiber optics is based on white-light interference [117], which can relate the absolute shifting of a signal emitted from a light source with any physical variable [118]. This type of sensor is used to measure deformation, temperature, material concentrations, acceleration, rotation, pressure, vibrations, and shifting. For deformation measurements, fiber Bragg grating (FBG) sensors and fiber optics sensors (FOS) are the most used. FBG sensors are used as selective filters of any wavelength [119], while FOS are composed of multimode fibers, have a low cost, and auto-compensate for temperature changes [120]. Deformation can be measured using three different approaches: (i) point sensors or discrete deformation that can locate the deformation [25]; (ii) quasi-distributed deformation sensors—an array of point sensors [121]—and (iii) distributed deformation sensors that can be used to determine a complete profile of deformations [122].

#### 4.1.5. Microelectromechanical Systems (MEMS)

This type of sensor uses miniaturization techniques in its construction [123], and different types of transducers can be combined. These sensors present advantages regarding the costs of implementation and maintenance [124]. It is also possible to take advantage of other attractive features [125], such as their small size [126] or their ability to easily connect to a wireless sensor network [127]. It is even possible to find sensors that use capacitive, inductive, piezoelectric, or optical effects [128,129]. In addition, actuators can be included [130]. In general terms, MEMS consist of the integration of different types of sensors [131].

MEMS are used to measure the magnitude of diverse variables, such as acceleration [127,132], angular velocity (gyroscopes) [133], displacement [134], and deformation [135]. This type of sensor offers high sensitivity [127], responses at low frequencies [136], the measurement of multiple variables, and the integration of communication systems [137]. Because of these factors, the use of MEMS has increased significantly. More precisely, several international research groups are developing a nanoelectromechanical system (NEMS) in an aim to increase the number of sensors in a structure and to thereby expand the analysis capabilities of existing SHM systems [138].

### 4.2. Location and Networking

The selection of an appropriate sensor not only depends on the measured variable but also must take into account aspects such as environmental and operational conditions, the number of sensors, the location of the network, and the energy consumption [139,140]. Figure 3 shows some of these aspects to be considered. The selection factors are divided into (a) sensor type; (b) operating conditions; and (c) limitations. In the first case, different elements, such as the variables to measure, the noise response, and the excitation method, are considered. Operating conditions refer to the interaction of the sensor with the structure during its operation. This means that the environmental and operational conditions must be considered when determining the approach to preprocessing the information and when defining the communication methods. Finally, limitations such as costs, implementation requirements, and setup of the sensor network must be considered.

Multiple works have been developed with the objective of determining the best way to locate and interconnect the network of sensors to be used. Decisions on these issues will define the success of data acquisition and will affect the cost of the instrumentation to use.

The work presented in Reference [141] produced a cost–benefit optimization method for the establishment of sensor networks by evaluating two metrics: an optimized benefit–cost ratio and maximized efficiency by complying with operational constraints. The method was applied to the instrumentation of the Pirelli tower in Italy.

### 4.3. Data Acquisition

The development of an inspection system depends on the way in which data related to the state of the structure are acquired. In this section, we review the works associated with the way in which excitation signals are generated as well as the selection of sensors; the location and communication of the sensors used; and the acquisition, storage, and transmission of data.

In general, the design of the monitoring system in terms of hardware starts from the definition of the sensor/actuator system—defined according to the previous section—with components that include piezoelectric transducers, microelectromechanical systems, optical fibers, or acoustic sensors [123]. After that, the design of the monitoring system should also take into account additional hardware that will be used to capture, store, and transmit the information. The ability to integrate the hardware in a large sensor network should also be assessed. Several works have addressed these topics. For example, an analysis of a method to determine an efficient sensor distribution in SHM was provided in Reference [143]. In Reference [144], the process of choosing wireless sensors for temperature and pressure measurements was described. In Reference [145], the authors reported a diagnosis related to the acquisition of information and the determination of the types of sensors to be used. Other works have analyzed the location of the sensor network [146,147], the preprocessing of information [148], and the interference of environmental conditions [149].

The general purpose of this step is to provide a signal to the following steps for the analysis of the state of the structure. It is desirable to comply with the requirements for precision, resolution, synchronization, and robustness to environmental and operating conditions [150]. This step is analyzed according to the main components of a data acquisition system: the signal conditioner, the digital–analog converter, and the transmission system.

Data acquisition systems need to be evaluated in the same way, depending on the location at which the information must be processed. This means that there is a difference between the assessment of a system that is required to process these data and the assessment of a system that is only required to capture, store, and transfer these data using different communication methods.

Some authors [20,151,152,153,154] have recommended that the following elements be considered for the selection of a DAQ system:

- the evaluation of the required number of inputs and outputs, that is, the number of digital or analog terminals to connect;
- the number of sensors;
- the need for an actuation system;
- the development of a quantified definition of the damage;
- the need for capturing local measurement or using remote sensing;
- the system-level responses—the information that is expected to be processed or preprocessed in the system;
- the possibility of implementing damage identification in an embedded system;
- the integration of feature extraction and statistical modeling algorithms with the sensing system;
- the consistent and retrievable archival of data for long-term monitoring;
- the transmission of information about the system condition to maintenance personnel or control systems;
- the operation of the sensing system with minimal maintenance over long periods of time in order to minimize the cost of the sensing and data acquisition system;
- the power consumption and source for long-term applications; and
- the evaluation of the operational and environmental conditions.

The above factors provide a complete picture of the type of sensor network that is required and the means of acquiring information from these sensor networks. The definition of the operating and environmental conditions to which the system will be exposed is also required: this has been a recurring topic for several authors [155]. This evaluation can reduce these factors when they are implemented by applying certain hardware elements or software strategies. These strategies include the use of interpolation and regression tools [156] to determine and then eliminate the influence of these variables [157]; the use of measurements that are independent of the influence of conditions that have been addressed using mathematical methods, such as singular value decomposition (SVD), PCA, auto-associative neural networks (AANN), factor analysis (FA), or cointegration; and, finally, the use of variables that are not affected in the short term by changes in environmental conditions [158], as is quite common in the instrumentation of civil structures.

### 4.4. Signal Conditioning

The measurement systems are exposed to several types of interferences that are produced by the monitoring system itself or by the environment in which the system is operating. Furthermore, the signals collected by the sensors may not have the voltage or power levels required for their analysis, or the signal delivered by the sensors may include unwanted noise in the subsequent analysis. Therefore, it is necessary to process these signals by amplification or attenuation, filtering, compensation, linearization, isolation, or compensation, among other signal-conditioning processes.

Depending on the type of sensor, it is possible to use different elements to satisfy the requirement of one of the steps mentioned above. When implementing signal-conditioning tools in SHM, the interoperability of all elements of the system should be reviewed. Some of the key features to be analyzed are the integration with the rest of the components and the increase in the success of the tool set. Similarly, the flow of information should be examined, and signal degradation, the output voltage levels, and the compatibility with analog-to-digital converters (ADC) should be considered.

In terms of the connectivity between the DAQ and processing devices, some systems offer more than one type of connection for cases of emergency. The following are some of the features that must be considered:

- *Expansion of the generated solution*: In many projects, the increment or adaptability of the created sensor network is required to increase the resolution or to decrease the range of values of a variable. The DAQ system must be able to adapt to these changes.
- *Isolation* should be considered in case of harmful signals for the processing schemes.
- *Bandwidth*: The information content of the sampled signal has to be transmitted with the fewest losses possible. This is achieved with the use of adequate sampling and the allocation of a suitable bandwidth.
- *Calibration* should be performed periodically to avoid failures in the processes of detection, location, or damage characterization.
- *Maintenance* is a relevant feature in continuous inspection systems since correct maintenance decreases the number of failures.

### 4.5. Preprocessing Step

Once data is acquired and preprocessed by hardware elements, it is possible to perform a preprocessing step, which is an important step for multivariate data analysis. This step allows to reduce random sources of variation in the data set. Since data in SHM can come from different kinds of sensors as was previously explained, there is not a general form to apply in all the cases; from this point of view, it is desireable to explore different methods to determine wich one produces the best results in the final damage-identification process. In general, this is obtained by determining the effectiveness of these techniques in terms of the measure of the separability of the evaluated groups and the accuracy of the classification of the pattern in each case.

These methods are not exclusively for SHM and have been applied in different areas where multivariate analysis is required. Among the main objectives of the preprocessing methods are noise removal, baseline removal, signal alignment, outlier detection, and data normalization. Some of the methods oriented to these aims are as follows:

- wavelet transform (continuous wavelet transform, discrete wavelet transform, and fast wavelet transform)[159,160];
- auto scaling [161,162];
- group scaling [163,164];
- variance scaling [17]; and
- Pareto scaling [165].

Information about each method can be found in the included references.

### 4.6. Data Reduction and Feature Extraction

The main objective of this step is to reduce the size of the data to analyse and to provide the features to be used in further analysis. Some of the techniques to be used in this step allow to transform the data in a new subspace or a representation by preserving the main features of the original data. Some of the techniques that can be used for feature extraction are as follows:

- Principal Component Analysis (PCA);
- Independent Component Analysis (ICA);
- Latent Sparse Domain Transfer (LSDT);
- Linear Discriminant Analysis (LDA);
- Fast Fourier Transform (FFT);
- Discrete Wavelet Transform (DWT) [166]; and
- Local Discriminant Preservation Projection (LDPP).

### 4.7. Prognosis Faults in SHM

**Figure 4:**
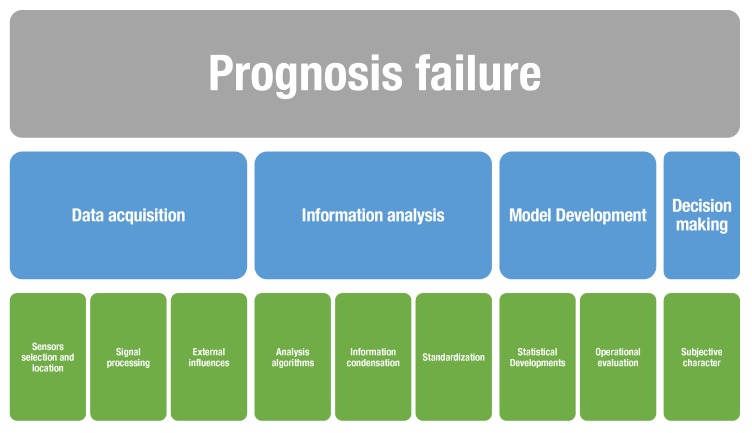
Prognosis failure approaches.

Fault- or damage-detection systems in SHM work on the basis of the quality of the information capture [167], the analysis of information, the use of statistical tools, and decision making (see Figure 4). If any of these steps are performed incorrectly, the fault- or damage-detection system may present errors that cause unnecessary interventions in the inspected structures (false alarms) or may fail to detect real damages in the structures [168] (missing faults).

Acquisition errors can result from the selection, location, configuration, or malfunction of the sensors. These failures imply relevant deviations between the signal obtained and the real value of the variable. The main causes of sensor failures are grouped into two types: *soft* and *hard* sensor faults. *Soft* sensor faults include bias, gain, loss of precision, and polarization. In contrast, *hard* sensor failures comprise constant deviations, deviations with noise, and failures due to background noise. In Reference [169], a mathematical analysis of the aforementioned faults was carried out.

Faults related to information processing occur in preprocessing and processing algorithms, condensation, and standardization [170]. Furthermore, the use of statistical tools can introduce errors to the analysis that, in some cases, lead to false detections. Finally, some errors can be identified in SHM that are associated with the subjectivity that is present in the analysis of the collected information. For instance, in multiple approaches, the levels of detection, location, and sizing include elements of subjective analysis, such as expert judgments.

In general terms, false detections are classified into two large groups: (i) *false positives*, also known as false alarms or type I errors, occur when the SHM system detects a fault or damage but the structure is healthy, and (ii) *false negatives*, also known as missing faults or type II errors, occur when the structure is faulty or damaged but the SHM system classifies it as healthy [170].

### 4.8. Development of Statistical Models

The use of statistical models has been well accepted in the development of damage-identification methodologies. Some of the works cited in the previous sections considered statistical models in their approach. As a complement to the works addressed above, some additional references are described in the subsequent paragraphs.

Some of these works have approached the fault- or damage-detection problem using multivariate analysis because of a large number of sensors or the use of a sensor network. One multivariate analysis method is PCA. This method has frequently been shown to be useful for data reduction [171], thus offering a great advantage in the analysis of data from sensor networks with a large number of sensor/actuator configurations [172]. However, PCA is only one of multiple methods used for this purpose. Some variations or alternatives include ICA [79,173] and DWT [166,174,175]. They are all very useful tools to re-express raw data in a new reduced version of the data while preserving the relevant information.

**Figure 5:**
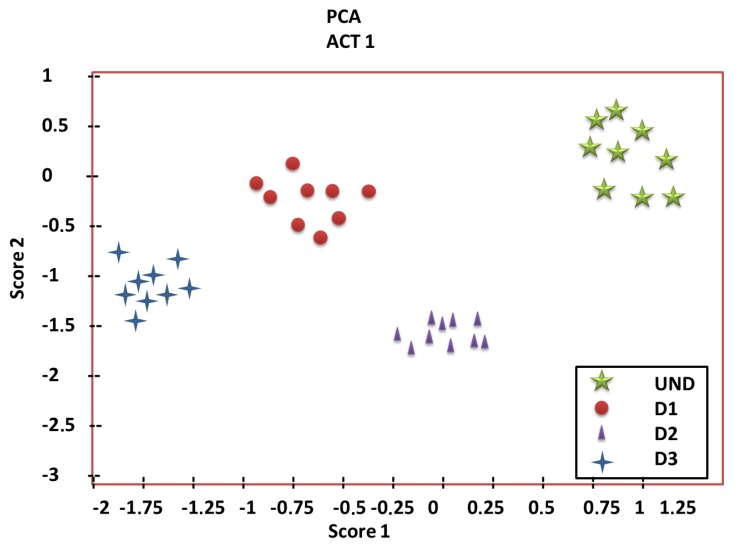
Score 1 versus Score 2.

More precisely, in damage detection, PCA can be used to change the original data by the corresponding projections of so-called principal components. From the perspective of pattern recognition, data from a healthy state of the structure can be used to define the *healthy pattern*. When the current structure has to be diagnosed, the new measured data can be projected into the PCA or ICA model to obtain so-called *scores*. Figure 5 represents the projection of the first two principal components of a data set in which no damage is present and with three different types of damage. As it is possible to observe that the data from the different structural states are very different; in this case, groups can be distinguished by simple inspection and damage detection can be applied. However, in most of the cases, these groups are mixed and cannot be differentiated in two axes.

The main differences between PCA and ICA are that components in ICA are linearly independent. Therefore, in the latter case, it is not possible to organize the components according to the proportion of the retained variance. This kind of plot is useful for visual analysis or inspection since data from the inspected structure can be analyzed and compared with the data from the healthy structure to determine the presence of damages. The presence of this damage is detected by the *visual* separation of the new data from the current structure to diagnose the data coming from the healthy structure. However, this methodology is useful only if the first two principal components retain a large proportion of the variance. In other cases, the data appear mixed, so it is not possible to detect and classify damages with a single visual inspection. In some of these cases, PCA can be combined with univariate and multivariate hypothesis testing to correctly classify the current state of the structure. Both univariate [3] and multivariate [176] hypothesis testing have been used for damage detection in small-scale structures as well as for fault detection in wind turbines [177,178,179].

Bayesian approaches have also been studied for damage detection [70] and impact detection. For instance, Morse et al. [69] applied a Bayesian updating (BU) approach and Kalman filter to estimate the location of impact. In general, BU provides a probabilistic prediction of the impact location, so, quantitatively, uncertainties associated with the prediction of the impact are permissible. Flynn and Todd [180] used a formulation of Bayes risk for optimal sensor and actuator placement using different kinds of sensors/actuators. The optimization space was searched by using a genetic algorithm with a time-varying mutation rate.

Anaya et al. [17,181] used both PCA and artificial immune systems (AIS) to detect damages in structures. In the proposed methodology, an active piezoelectric system is used to inspect the structure and to collect the data. The information is encoded by emulating the effect of human immune systems [182] and by considering external elements to be possible damages. This approach defines antibodies as the *detector* of a specific pattern and antigens as the damage condition.

**Figure 6:**
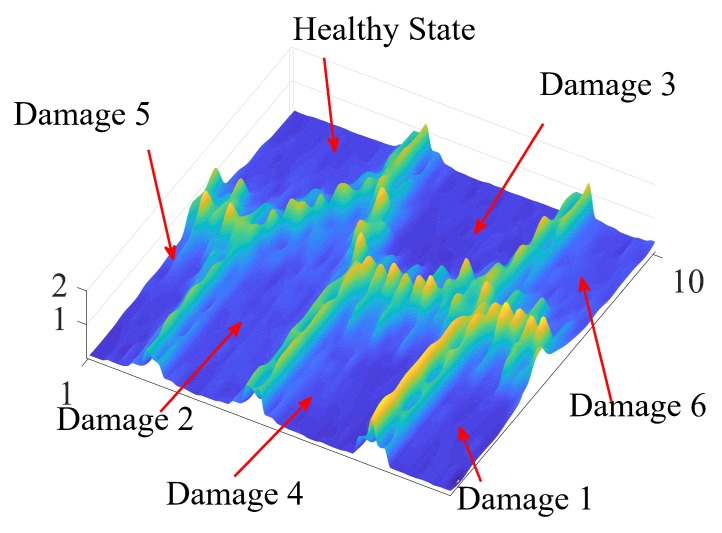
U-matrix surface.

The use of SOM has also been applied for damage detection and classification [183]. SOM are unsupervised neural networks in which training is a blind process that enables the grouping and classification of different kinds of data according to data features. In general, in this kind of methodology, data reduction is performed using techniques such as PCA or DWT and the new components, coefficients, or indices are used as a feature vector; all the results from all the actuation phases are fused into a single vector. This vector acts as the input to train the self-organizing map. When the pattern is built or defined, the same procedure is applied to the structure being tested to evaluate the state of the structure and to determine the presence of damages and their possible classification. As a visualization tool, it is very common to use a cluster map or a U-matrix surface [64]. Figure 6 shows an example of the use of the U-matrix surface; seven structural states are considered in the illustrated classification process: specifically, one healthy structural state and six different damages are included in this plot. Since the methodology requires previous training, the algorithm considers new data with features that differ from those of the data used during training to be a new cluster or a new type of damage.

Machine-learning approaches have also been studied, on their own or in combination with different feature extraction methods [184], as damage detection and classification methodologies. In this kind of approach, in general, PCA is used as a feature-extraction method to define the feature vector to train the machine using several states of the structure. During the test step, data from an unknown structural state are evaluated by the trained machine, and the classification can be performed. Algorithms that are commonly used in machine learning include *k*-nearest neighbor (kNN), decision trees, and support vector machines (SVM).

The Gaussian process has also been studied [76] for probabilistic data-driven modeling and structural damage detection methods. In general, data from the structure to be inspected and instrumented with sensors, such as piezoelectric transducers working in pitch-catch mode, provide information that can be pre-processed using a DWT. The details and coefficients of the DWT are subsequently used for the extraction of features, which are simultaneously the input of the Gaussian process. The output of this training—that is, the first step—is the corresponding pattern. In the second step, data from the tested structure are pre-processed as in the first step. Afterward, the features are included in the Gaussian process and a comparison with this pattern is obtained for the classification process. The validation of the methodology can be performed using receiver operating characteristic (ROC) curves to evaluate the effectiveness of the classification.

Nonlinear approaches, such as nonlinear PCA (NLPCA), have also been analyzed for damage detection and classification. Some examples include the combination of the hierarchical version of NLPCA (h-NLPLCA) and machine learning, in which the nonlinear components are used as the feature vector to train different models [185].

Damage progression has also been addressed with the use of piezoceramic arrays [186]: a number of time- and frequency-domain features are derived from existing damage imaging and detection algorithms combined with data-mining algorithms.

**Figure 7:**
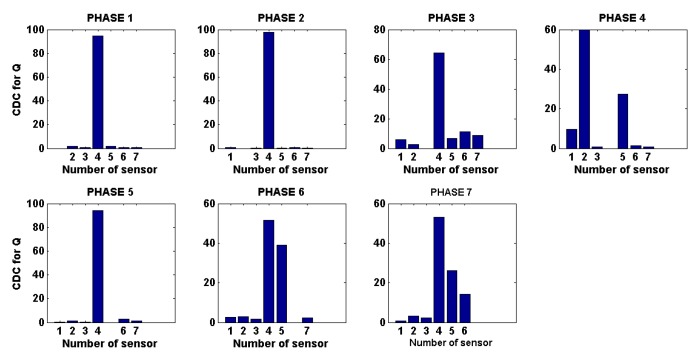
Damage contribution index.

In terms of damage localization, damage indices have also been explored [32] as damage indicators and applied to complement PCA to reduce the problems that arise when the two first principal components do not retain a significant proportion of the variance. Two of these indices are the *Q*-index and the $T^{2}$-index, which can be used to determine the contribution of a specific sensor in the sensor network. Subsequently, using triangulation techniques, the potential area or the position of the damage can be defined. One example of the kinds of diagrams that are obtained by the use of contribution plots is shown in Figure 7. This figure shows an example with seven actuation phases, and each phase shows the contribution of each sensor in the sensor network. In this way, localization can be performed by triangulating the sensors with the highest contributions and the piezoelectric used as actuator.

These indices have also been used in damage detection to process data from FBG sensors [187] with good results. Hierarchical clustering (HC) is another supervised method of statistical learning that is commonly used in SHM. As the name suggests, this algorithm collects and groups similar data using a set of distance measures that define the similarity of the data. Commonly, at the detection level, the established parameter is represented by a damaged or undamaged state. HC usually builds a hierarchy of data groups and uses a different function to assign data points to groups. The Euclidean distance and Mahalanobis distance are two popular choices among many that are used for a dissimilarity function [188].

HC has been applied at the detection level for several types of structures. Datteo et al. [189] proposed an HC analysis to obtain a set of clusters based on damage patterns that were found in the experimental data, which were collected from PZT sensors by means of a graphic representation of the information so that the damage could be identified intuitively, both qualitatively and quantitatively. The performance of the proposed approach was tested by three experiments on a full-scale reinforced concrete beam. In another work, Zhou et al. [190] incorporated HC with a method for recognizing artificial immune patterns for the recognition and classification of damage patterns in the health monitoring of an unsupervised structure. The sampled data were classified by an agglomerated hierarchical clustering algorithm. Then, sets of memory cells were trained to imitate the mechanism of learning and immunological recognition. Finally, structural data patterns were judged by the sets of trained memory cells. The results of this work showed that agglomerated hierarchical clustering and incorporated methods could successfully identify the most patterns in the antigen sample data. The work concluded that the unsupervised structural damage-classification algorithm based on HC and artificial immune patterns could produce high-quality memory cells that could effectively identify all types of structural damage patterns. Finally, Sen et al. [188] proposed two data-based techniques—a semi-supervised and supervised learning approach—for the detection of damage in pipelines. The proposed approaches aimed to reduce the number of sensors deployed and to thus reduce maintenance costs. The semi-supervised learning method detected the presence of damages using an algorithm based on hierarchical grouping, and the approach based on supervised learning located damages using multinomial logistic regression. The proposed algorithms were validated by the acquisition of guided ultrasonic wave (GUW) responses from experimental pipelines in a tone capture configuration using low-cost piezoelectric transducers.

Unsupervised statistical methods of learning are also widely used in SHM analysis [191]. One of the reasons is that the algorithms involved are less complex, so they can be used in real-time analysis [192]. These methods were compared with traditional alternatives in works such as the one carried out by McCrory et al. [193], in which three classification techniques were tested—analysis of artificial neural networks (ANN), unsupervised waveform clustering (UWC), and the corrected measured amplitude ratio (MAR)—in the location and classification of faults in a composite panel of carbon fiber during buckling. This paper reported that UWC and ANN were better classifiers and that their use improved the reliability of the SHM system. Nagarajaiah et al. [194] applied unsupervised methods for the analysis of structures under vibration conditions. The authors used multivariate blind source separation to detect anomalies, and they reported positive results. Despite the stated advantages, unsupervised learning methods are not implemented to the same extent as supervised methods. One reason is the reliability of the obtained results.

Uncertainty quantification has been examined in different fields. One example is the inspection of cultural heritage [195]: in this study, uncertainty reduction was applied to the modal estimation of data from two historical buildings. In Reference [196], uncertainty quantification in OMA was developed for vibration-based analysis. Structural excitations were not directly measured but rather modeled by band-limited white noise processes.

The classification process can be regarded as a binary classification problem. One example is the work in Reference [197], in which possibility theory was introduced to solve a decision-making problem involving conflicting information. The information was modeled as weighted intervals on the basis of importance, and a possibility distribution from the weighted intervals was presented to fuse information with respect to its importance.

### 4.9. The Decision Level

Decision support systems (DSS) have been used extensively in the analysis of economic, technical, environmental, optimization, and other problems that involve a choice of alternatives [198]. This type of tool is an important component in monitoring and control systems. However, there are few studies on this topic in relation to SHM systems, and most of these works have focused on the monitoring of civil structures.

The research efforts on this matter have involved the implementation of different decision tools to assess the state of the structure. Endsley et al. [199] integrated information from the data banks of the National Bridge Inventory (NBI) with measurements taken from various bridges using nondestructive evaluation (NDE) systems. They unified the information and presented a web application to improve the decision-making processes and to reduce the subjectivity in the interpretation of the data. Sun et al. [200] presented a hybrid system that combined neural network theory and adaptive fuzzy logic to generate a framework for the analysis of heterogeneous signals from various types of sensor networks. The implementation of the hybrid system enabled the evaluation of the type of study to be carried out on each data source in the monitoring scheme. SHM analysis has been used as a test scenario for decision models; Khodaei et al. [201] compared expected utility theory (EUT) with prospect theory (PT) in the estimation of the state of a bridge. Their work showed that the opinions on inspected structures varied because of the subjectivity of the person who analyzed the results.

Different works present proposals related to the management of decisions and decrease of detection failures by defining threshold levels; the work presented in Reference [202] summaries the use of different categories of SHM in the analysis of bridges and civil constructions. This work presents an interesting summary of implementation techniques in the category of SHM implementation related to the definition of limits in the analyzed variables; among them, humidity, vibration, and settlement are found, emphasizing the probabilistic uncertainty quantification models. Bai et al. [203] present a paper oriented to the reduction of false forecasts by evaluating three techniques that use the information generated by fatigue in structures instrumented with Acoustic Emission (AE) sensors. Results of this work show the influence on the definition of threshold levels in the automation of decisions. Deraemaeker et al. [204] present a proposal for decreasing the influence of environmental conditions using filters and definitions of distances on covariance matrices and eigenvalues. As a result of this approach, the high sensitivity of the measurement systems and the efficiency of the definition of thresholds in the reduction of false positives is presented; this study was carried out in civil structures. In Reference [205], a laboratory test is applied to a metallic structure instrumented with piezoelectric transducers in a system based on electromechanical impedance; a part the analysis shows that threshold levels significantly influence the detection and location of faults.

Very few works on the development of DSS have examined the monitoring of composite and metallic structures. One such work was carried out by Bolognani et al. [206], who implemented a multilevel decision scheme to reduce the costs of processing and instrumentation. The generated method could reduce the risk of errors in evaluation (false positive and negative), increase the profitability of the instrumentation, and unify the results of different information sources. Finally, the work reported by Sabatino et al. [207] focused on the development of a framework that aimed to improve the cost–benefit in the choice of sensing schemes for naval structures. They reported that their framework could decrease the risks associated with choosing the data-processing system to be used.

From the existing studies, a decision level for the analysis of composite materials is presented that would be located in the upper part of Figure 2. The function of this level is focused on the choice and evaluation of the types of processing of data from structures instrumented with different types of sensors.

## 5. Conclusions

In this review, several elements of the SHM process and its implementation are explored. These elements are the description of the SHM process and the components of SHM implementation, including sensors and actuators, location and networking, data acquisition, signal conditioning, statistical analysis, and decision levels. It is shown that diverse authors have made significant contributions to these achievements. From the presented information, the following general conclusions are drawn:

- The works related to the advances and implementation of SHM systems account for the monitoring requirements of healthy structures in diverse areas of applications, such as civil engineering, aeronautics, transport, and power generation. This research field, which is still developing, presents research opportunities related to methods for sensor selection and location, communication systems, analysis of environmental and operational conditions, reduction of false positives and false negatives, and decision methodologies, among others.
- There has been a considerable increase in the use of SHM systems in operating structures. This increase, together with the emergence of regulations for the operation of systems for monitoring structures, reflects the rapid adoption of SHM in industries such as the automotive and aeronautics sectors and intelligent materials development. As a result, a significant growth in investment, leading to the application of all levels of SHM, is expected.
- Although this paper presents the steps of implementing an SHM system, these steps should be used only as a reference: they can be decomposed or complemented according to the implementation and the intended approach. This is currently a focus that is actively researched to improve the reliability of the elements of the implementation.
- Using data that are directly acquired from sensors installed on the structure is a convenient way to evaluate the current state of a structure. This allows for the continuous measurement of data to monitor applications in real time. However, some problems may arise during the implementation of these SHM approaches. Such challenges include the following:
  (a) Failures in the sensors are possible and can arise from problems during installation or damages to the sensor when the structure is subjected to hard conditional and operational variations. This problem can be solved by the use of faulty sensor detection, similar to damage detection. In some cases, it is possible to reconstruct the signal to avoid false damage detections during the damage-identification process. Similarly, there are some data-driven algorithms that can compensate for the effects of environmental and operational variations; these are required because, as it was explained, environmental and operational variations can change the baseline and can produce false positive damage identification.
  (b) Poor use of preprocessing techniques often leads to poor results of the damage-identification process. This problem can be partially solved by the design of robust methodologies.
  (c) Some problems are related to storing data and processing the information coming from large structures instrumented with a large number of sensors. In some cases, such problems can be easily solved through a distributed analysis.

Although there are several proposed solutions to the different problems in the task of identifying damage, most of the results are relevant to a specific application and are tested under laboratory conditions. Hence, problems at different levels of the damage-identification process, such as the problems associated with data acquisition and preprocessing, remain open for investigation.

According to the number of publications, one of the least explored topics is the implementation of decision support tools. This document demonstrates the need for such tools in data-driven applications. Some studies have focused on specific cases, but the different alternatives of all the levels explored require an intelligent system that is able to decrease the number of false positives and negatives in the identification process. Its development and implementation will allow for the mixing of different types of information sources and for eliminating the subjectivity of the analysis, among other improvements.

## Figures and Tables

**Table 1:** Sensor types and uses.

Sensor Type	Technology	Variable to Measure	Advantages	Disadvantages	Relevant Features
Piezoelectric	PZT	Acceleration	Low cost	Thermal sensitivity	Used in EMI applications
	PVDF	Deformation [106]	Low price	Aging	Wide range of frequencies [142]
	P(VDF-TrFE)	Corrosion [107,108]	Integration possibilities		Shape adaptation [109]
		Displacement			Reduced phase
		Vibration			changes [112]
Fiber optics	FBG	Deformation [122]	High precision	High price	
	FOS	Acceleration [119]		Fragility	
		Rotation	Electromagnetic interference immunity [117]		
		Pressure			
		Vibrations	Integration possibilities		
		Shifting			
Microelectromechanical systems (MEMS)	MEMS	Deformation	Low cost [124]	High-frequency response [136]	
	NEMS [138]	Acceleration [127,132]	Small size [126]	Fragility	
		Gyrometer			
		Displacement [134]	Wireless connection [127]		
		Deformation [135]	Different kinds of sensors and variables [131]		
		Shifting			

## Abbreviations

The following abbreviations are used in this manuscript:

AANN	Auto-associative neural networks
ADC	Analog-to-digital converters
AE	Acoustic emission
AIS	Artificial immune systems
ANN	Analysis of artificial neural networks
BF-SHM	Baseline-free SHM
BHM	Bridge Health Monitoring
BU	Bayesian updating
DAQ	Data acquisition
DSS	Decision support systems
DWT	Discrete wavelet transform
EMI	Electromechanical impedance
EMIS	Electromechanical impedance spectroscopy
EUT	Expected utility theory
FA	Factor analysis
FBG	Fiber Bragg grating
FDD	Frequency domain decomposition
FEA	Finite element analysis
FOS	Fiber optics sensors
GUW	Guided ultrasonic wave
HC	Hierarchical clustering
h-NLPLCA	Hierarchical nonlinear principal component analysis
ICA	Independent component analysis
IoT	Internet of Things
kNN	*k*-nearest neighbor
LDV	Laser Doppler vibrometer
LVDT	Linear variable differential transducer
MAR	Measured amplitude ratio
MEMS	Microelectromechanical systems
ML	Machine learning
NBI	National Bridge Inventory
NDE	Nondestructive evaluation
NEMS	Nanoelectromechanical system
NLPCA	Nonlinear principal component analysis
OMA	Operational modal analysis
PCA	Principal component analysis
PDI	Probability damage imaging
PT	Prospect theory
PVDF	Polyvinylidene difluoride
P(VDF-TrFE)	Poly(vinylidene-co-trifluoroethylene)
PZT	Piezoelectric transducer
RF	Radio frequency
RMSD	Root-mean-square deviation
ROC	Receiver operating characteristic
SFEM	Smoothed finite element method
SHM	Structural health monitoring
SOM	Self-organizing maps
SVD	Singular value decomposition
UWC	Unsupervised waveform clustering
